# Disrupted progression of the intestinal microbiota with age in children with cystic fibrosis

**DOI:** 10.1038/srep24857

**Published:** 2016-05-04

**Authors:** Shaun Nielsen, Bronwen Needham, Steven T. Leach, Andrew S. Day, Adam Jaffe, Torsten Thomas, Chee Y. Ooi

**Affiliations:** 1 Centre for Marine Bio-innovation, University of New South Wales, Sydney, 2052, Australia; 2 School of Biotechnology and Biomolecular Sciences, and University of New South Wales, Sydney, 2052, Australia; 3 Discipline of Paediatrics, School of Women’s and Children’s Health, Medicine, University of New South Wales, Sydney, 2052, Australia; 4 Department of Paediatrics, University of Otago, Christchurch, 8011, New Zealand; 5 Department of Paediatric Respiratory and Sydney Children’s Hospital, Randwick, 2031, Australia; 6 Department of Paediatric Gastroenterology, Sydney Children’s Hospital, Randwick, 2031, Australia

## Abstract

Cystic fibrosis (CF) is a genetic disorder that leads to formation of thick epithelial secretions in affected organs. Chronic microbial infections associated with thick mucus secretions are the hallmarks of lung disease in CF. Despite similar conditions existing in the gastrointestinal tract, it is much less studied. We therefore examined the microbial communities within the gastrointestinal tract of children with and without CF (either pancreatic sufficient or insufficient) across a range of childhood ages (0.87–17 years). We observed a substantial reduction in the richness and diversity of gut bacteria associated with CF from early childhood (2 years) until late adolescence (17 years). A number of bacteria that establish themselves in the gut of healthy children were unable to do so in children with CF. In contrast, a few bacteria dominated the gut microbiota in children with CF and are unlikely to be beneficial for the metabolic function of the gut. A functioning pancreas (pancreatic sufficient) under a CF lifestyle showed little effect on microbial communities. Our results argue that any attempts to rectify the loss of bacterial diversity and provide normal bacterial function in the gut of CF patients should be conducted no later than early childhood.

Cystic fibrosis (CF) is an autosomal recessive disorder associated with mutations in the gene coding for the cystic fibrosis transmembrane conductance regulator (CFTR) protein^1^. The CFTR protein functions on the apical surface of epithelial cells in the airways, pancreas, intestines and hepatobiliary tree as an anion-selective ion channel (mainly chloride and bicarbonate), and thus contributes to epithelial fluid secretion and intra-luminal mucus hydration^2^. Among all the different organ systems affected, the exocrine pancreas is the most reliable phenotypic barometer of the degree of CFTR dysfunction^3^,^4^. Most patients carrying severe mutations (i.e. Class I–III) on both alleles have a pancreatic insufficient (PI-CF) phenotype, while patients who carry a mild mutation on at least one allele, and thus have residual CFTR function, are usually are pancreatic sufficient (PS-CF)^3^,^4^,^5^.

Microbial colonization and infections due to impaired airway clearance from thick mucus secretions are the hallmarks of CF lung disease. Similar conditions that could contribute to the development of dysbiosis (microbial imbalances) also exist in the gastrointestinal tract (the gut) of CF patients. Patients with CF have reduced bicarbonate secretion from the pancreas, intestines and biliary tree as part of the primary CFTR defect. The lack of bicarbonate results in increased luminal viscosity due to formation of inspissated mucus in the intestinal tract^6^ as well as a more acidic small intestinal environment^7^. Regular use of antibiotics due to recurrent pulmonary infections, increased load of malabsorbed luminal contents and impaired innate immunity^8^,^9^ may further contribute to the development of microbial dysbiosis in the gut.

Intestinal bacterial overgrowth has been long recognized as a feature of CF^10^, but the recent availability of culture-independent approaches to characterise the diversity and responses of microbial communities has led to emerging interests in the gut microbiome associated with CF^11^,^12^,^13^,^14^,^15^. The gut microbiome consists of complex communities, which significantly enrich the metabolic capacity of humans^16^ and have drastic effects on host physiology and immunology and thus also on human health and wellbeing^17^. Alterations in the gut microbiome have also been speculated to play a role in the development of intestinal inflammation observed in CF^11^,^18^,^19^,^20^,^21^. The importance of the gut microbiome in CF may also extend beyond the gut. Gut colonization patterns have been speculated to play a role in the development of the respiratory microbiota^22^ and cirrhosis in CF^20^.

Significant differences in the diversity and composition of the gut microbiome have been observed between healthy and CF individuals in humans^11^,^12^,^13^ and mice^23^. In all cases, the gut microbial communities within the CF population had reduced abundances of specific bacterial taxa including members of the genera *Clostridium*, *Eubacterium*, *Feacalibacterium*, and *Bacteroides* as well as the order Lactobacilliales. There is emerging evidence that such dysbiosis within the CF gut can facilitate the growth of opportunistic pathogens such as potentially pathogenic *Escherichia coli* and *Eubacterium biforme*^24^. Temporal changes have been less explored, but succession of microbial communities is a known phenomenon within different environments associated with the human body, including the infant gut^25^, and could be influenced by environmental stressors such as the acidic and inspissated intestinal secretions in CF^26^.

Given the current knowledge, there is still a lack of understanding of the temporal changes of the gut microbiome within the CF population and how these compare with temporal changes seen in healthy (non-CF) children. The effects of different severity of CFTR dysfunction on these changes have also not been evaluated. In this study, we therefore examined the microbial communities within the gastrointestinal tract of children with and without CF. We aimed to understand changes in microbial diversity and composition in the setting of CF, and how these changes manifest themselves on a temporal scale within patients across a broad age range in childhood. As a secondary aim, we evaluated the effect of exocrine pancreatic status, as a surrogate for the severity of CFTR dysfunction, on the gut microbiome.

## Results

### Rarefaction analysis

After 16 S rRNA gene sequence quality filtering, rarefaction curves detailing the number of OTUs as a function of sequencing sampling depth reached saturation at smaller sampling depths for the CF cohort compared with heathy cohort (HC, Supplementary Figure 1a). Similarly, curves of the pancreatic sufficient and insufficient cohorts (PS-CF and PI-CF, respectively) reached saturation earlier than the HC cohort (Supplementary Figure 1b). Overall, the rarefaction analysis indicated a sufficient coverage of the microbial communities with the given 16 S rRNA gene sequencing effort.

### Alpha-diversity of gut microbiomes between healthy and CF cohorts

**Figure 1:**
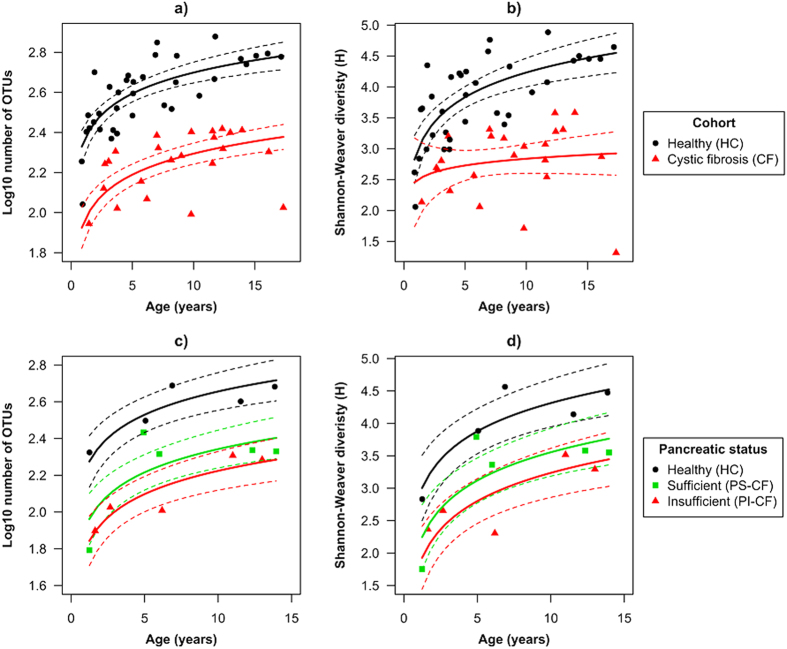
The relationship of microbial richness and diversity (Log10 number of OTUs, (**a**,**c**)) and diversity (Shannon-Weaver index, (**b**,**d**)) with age within gut microbiome cystic fibrosis (CF) and healthy children (**a,b**), or children with CF and pancreatic sufficiency or insufficiency (**c,d**). Fitted lines and 95% confidence intervals are constructed from general linear models (for **a,b**) n_HC_ = 35 and n_CF_ = 23, and for (**c,d**) n = 5 for each cohort).

There were clear trends in OTU richness and OTU diversity (Log10 number of OTUs and Shannon-Weaver index, respectively) associated with age and between cohorts (Fig. 1a,b). Generally, the number and diversity of OTUs increased with age for both cohorts, but was consistently lower in the CF cohort compared with the HC cohort. The adjusted mean (i.e. the mean adjusted for the effect of age) of the Log10 number of OTUs for the CF cohort was 2.21 (95% CI: 2.15–2.26) and significantly lower compared with 2.61 (95% CI: 2.57–2.65) of the HC cohort (ANOVA–F_1,54_ = 138, P = 2.2 × 10^−16^). Every doubling in age (Log2 transformed) was associated with a 0.11 (95% CI: 0.08–0.15, ANOVA–F_1,54_ = 51, P = 2.2 × 10^−9^) increase in the Log10 number of OTUs for both cohorts, with the mean Log10 number of OTUs in the control and CF cohorts 2.35 and 1.94, respectively, at age = 0 (Fig. 1a). The species richness in the CF cohort, even at 15 years of age, did not reach the same richness found in HC cohort at one year of age (Fig. 1a). Intra-subject variability of the Log10 number of OTUs of seven CF subjects was well within the variability of the CF cohort (Supplementary Figure 2a).

The adjusted mean of the Shannon-Weaver index was significantly lower in the CF than HC cohort and was 2.75 vs. 3.90 (95% CI: 2.51–2.98 and 3.72–4.08), respectively (ANOVA–F_1,54_ = 70, P = 2.5 × 10^−11^). Furthermore, the size of the difference between the cohorts strongly depended on age (ANOVA - Age x Condition: F_1,54_ = 4.6, P = 0.036, Fig. 1b). Every doubling in age was associated with a 0.40 (95% CI: 0.25–0.55) increase in the Shannon-Weaver index for the control cohort (from 2.9 at age = 0), but only 0.11 (95% CI: −0.31–0.53) for CF cohort (from 2.4 at age = 0, Fig. 1b). These results showed that the CF cohort had constantly low species diversity, while a substantial increase with age was observed in HC (Fig. 1b). The microbial diversity in CF patients never reached that found in the HC group and most of the differentiation appeared to have occurred in the first three years of life. Intra-subject variability of the Shannon-Weaver index of seven CF subjects was well within the variability of the CF cohort (Supplementary Figure 2b).

### Alpha-diversity of gut microbiomes between pancreatic sufficiency cohorts

Both alpha-diversity measures showed differences associated with the exocrine pancreatic status and trends with age (Fig. 1c,d). In the analysis comparing five HC, PS-CF and PI-CF subjects each, a gradation in the mean values of alpha-diversity measures was observed according to the exocrine pancreatic status i.e. HC > PS-CF > PI-CF. Similar increasing trends with age occurred within each cohort.

The adjusted mean of the Log10 number of OTUs was 2.12 (95% CI: 2.01–2.23) for the PI-CF cohort, 2.23 (95% CI: 2.13–2.34) for the PS-CF cohort and 2.55 (95% CI: 2.44–2.67) for the HC cohort (ANOVA–F_2,9_ = 18, P = 6.8 × 10^−4^). Every doubling in age (Log2 transformed) was associated with a 0.13 (95% CI: 0.08–0.18) increase in the Log10 number of OTUs for all cohorts, with the mean Log10 number of OTUs 1.80, 1.92 and 2.23 at age = 0 for the PI-CF, PS-CF and HC cohorts, respectively. Given the gradation between cohorts, the PS-CF cohort was more comparable to the PI-CF cohort than the HC cohort based on 95% confidence intervals (Fig. 1c,d).

The adjusted mean of the Shannon diversity index, in increasing order, was 2.11 (95% CI: 2.01–2.22) for the PI-CF cohort, 2.24 (95% CI: 2.13–2.2.34) for the PS-CF cohort and 2.55 (95% CI: 2.44–2.66) for the HC cohort (ANOVA–F_2,9_ = 8.7, P = 0.008). Every doubling in age was associated with a 0.43 (95% CI: 0.25–0.62) increase in the OTU diversity for all cohorts, with the mean Shannon’s diversity 1.79, 2.11 and 2.87 at age = 0 for the PI-CF, PS-CF and control cohorts, respectively. As with the richness patterns observed above, the PS-CF cohort was more comparable to the PI-CF cohort than the HC cohort based on 95% confidence intervals (Fig. 1c,d).

### Beta-diversity of gut microbiomes between healthy and CF cohorts

**Figure 2:**
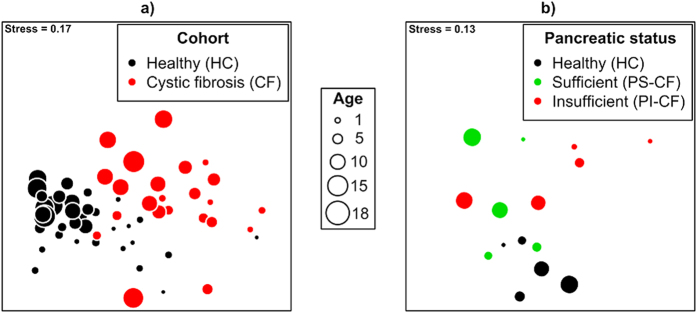
Non-metric multidimensional scaling (nMDS) ordination of gut microbiome communities within (**a**) CF and healthy children, and (**b**) children with CF and pancreatic sufficiency or insufficiency, compared using the Bray-Curtis similarity coefficient of square-root transformed OTU relative abundances. Symbols represent samples, and distances between symbols represent similarities between samples (closer symbols are more similar than distant symbols). Symbol size is proportional to age. The number of samples for (**a**) are n_HC_ = 35 and n_CF_ = 23, and for (**b**), n = 5 for each cohort.

Comparison of the beta-diversity among HC and CF cohorts revealed that all samples shared, on average, 22.4 ± 9.7% similarity (Bray-Curtis units of square-root transformed OTU relative abundance). Similarities between samples within cohorts were 26.9 ± 9.5% and 23.6 ± 7.5% for the HC and CF cohorts, respectively. Visualisation of the similarities between samples revealed separation of samples between cohorts and that within the control cohort samples became similar with increasing age (Fig. 2a). There was evidence of an interaction between associated with age and cohorts on Bray-Curtis similarities (PERMANOVA: F_1,54_ = 1.67, P = 0.034) confirming clustering patterns and affirming that age related compositional trajectories were affected by the CF condition.

### Beta-diversity of gut microbiomes between pancreatic sufficiency cohorts

Comparison of the beta-diversity among HC, PS-CF and PI-CF cohorts revealed that all samples shared, on average, 20.4 ± 8.8% similarity. The HC cohort shared 30.7 ± 6.5% similarity, which was greater than that of 20.4 ± 7.3% and 22.4 ± 9.4% observed in the PS-CF and PI-CF cohorts, respectively. Visualisation of the similarities between samples revealed clustering of samples within the healthy cohort and weak clustering of samples within PS and PI cohorts (Fig. 2b). Changes in community composition with age differed among the three cohorts (PERMANOVA–Age x PS condition: F_2,9_ = 1.37, P = 0.05), with greater variability between samples for the PS-CF and PI-CF cohorts compared with the healthy cohort (Fig. 2b). In light of this result, there were differences, irrespective of age, between the three cohorts (ANOVA–PS condition: F_2,9_ = 1.86, P = 0.001), principally due to the difference of the HC cohort with the other two cohorts (PERMANOVA pairwise comparisons, all P < 0.03).

### Comparison of OTUs between communities in healthy and CF cohorts

**Figure 3:**
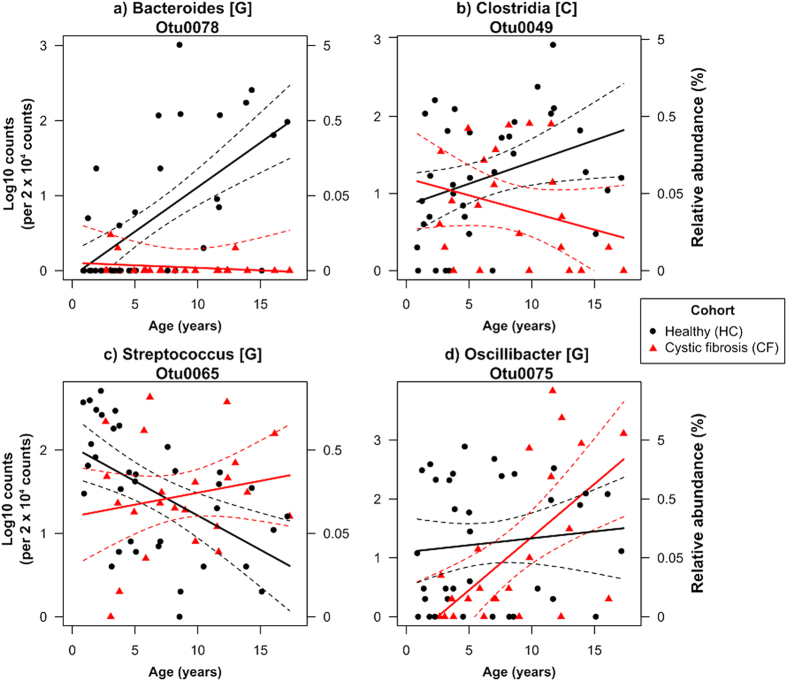
Different relationship types between age and the abundance of OTUs in gut microbiomes communities from CF and healthy children (HC). Representative OTUs show abundance trends with age that are (**a**) positive in HC, but absent in CF, (**b**) opposite between HC and CF, (**c**) negative in HC, but constant in CF and (**d**) positive in CF, but constant in HC. Fitted lines and 95% confidence intervals are constructed from general linear models (n_HC_ = 35 and n_CF_ = 23). Letters in square brackets following taxonomic names within titles denote the lowest level of taxonomic classification: G = genus, C = Class.

After removal of rare or ambiguous OTUs, the abundances of 107 OTUs were modelled by age and cohort. Multiple testing adjustment severely inflated P values, thus we focused on test statistics (F values) in parts of the analysis (F values > 5, where P ~ 0.02 without adjustment given the degrees of freedom) in order to not disregard too much data. Fifteen OTUs showed differential abundance trends between the two cohorts and age (Age x Cohort, F_1,54_ > 5, Supplementary Figure 3, a subset of the four trend types observed are shown in Fig. 3). Eleven OTUs showed increases in abundance with age in the HC cohort, but were consistently low in abundance among the CF cohort (an example shown in Fig. 3a). These OTUs belonged to unclassified genera within the families *Ruminococcaceae* and *Lachnospiracea*e (both phylum Firmicutes) and the genera *Oscillibacter* (family *Ruminococcaceae*), *Coprococcus* (family *Lachnospiracea*e), *Alistipes* (phylum Bacteroidetes). An OTU belonging to the class *Clostridium* (phylum Firmicutes) was near equal abundance between the two cohorts, but had opposites trends with age (Fig. 3b). Another two OTUs from the genera *Streptococcus* and *Falvonifractor* (both phylum Firmicutes) showed decreasing abundances with age in the HC cohort, but were consistently abundant in the CF cohort (*Streptococcus* OTUs shown in Fig. 3c). Lastly, an OTU from the genus *Bacteroides* (phylum Bacteroidetes) showed an increasing abundance with age in the CF cohort, while showing a neutral trend in the HC cohort (Fig. 3d).

**Figure 4:**
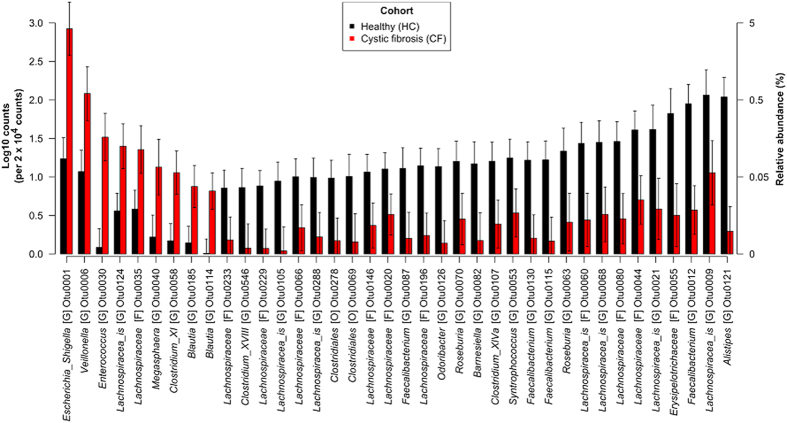
Differentially abundant OTUs in gut microbiomes communities from CF and healthy (HC) children (not showing interactions between age and CF condition). Adjusted means and 95% confidence intervals constructed from general linear models (n_HC_ = 35 and n_CF_ = 23). Letters in square brackets following taxonomic names denote the lowest level of taxonomic classification: G = genus, F = family, O = order. The suffix “*_is”* = *incertae sedis*.

Thirty-one OTUs were significantly less abundant in CF disease compared to HC controls (P_adjusted_ < 0.05) regardless of age. Within the CF cohort, the adjusted means of these OTUs were 0.02–0.3 times the abundance of that observed in the HC cohort (Fig. 4). Each of these OTUs typically represented small proportions in the communities of the HC cohort ranging from 0.04–0.6% of the total abundance (together 4% of the total abundance), and decreased to 0.005 to 0.05% of the total abundance communities of the CF cohort (0.4% when combined). Most OTUs were associated with a diverse range of genera from the families *Ruminococcaceae* and *Lachnospiraceae* (phylum Firmicutes) and there were also representatives from the genera *Barnesiella*, *Odoribacter* and *Alistipes* (phylum Bacteroidetes, Fig. 4).

**Figure 5:**
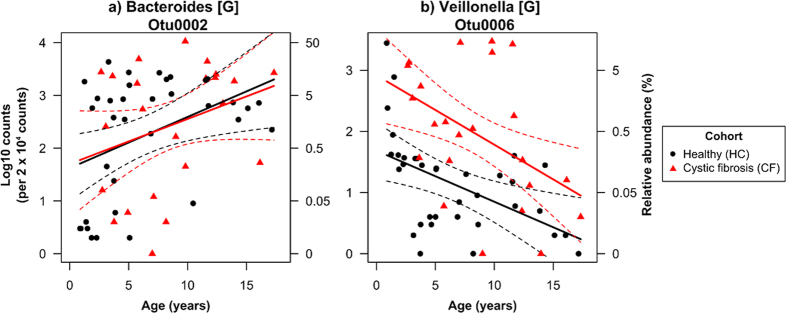
Relationships between age and the abundance of OTUs in gut microbiomes communities from CF and healthy children (not showing interactions between age and CF condition). Representative OTUs show abundance trends with age that are similar between both cohorts, despite differences between cohorts (seen in b). Fitted lines and 95% confidence intervals are constructed from general linear models (n_HC_ = 35 and n_CF_ = 23). Letters in square brackets following taxonomic names denote the lowest level of taxonomic classification, G = genus.

Only nine OTUs were significantly more abundant (P_adjusted_ < 0.05) in the CF cohort compared with the healthy cohort (Fig. 5). Within the CF cohort, these OTUs were 5.4–49 times that of the abundance observed in the HC cohort. Each of these OTUs typically represented 0.005–0.09% of the total abundance in communities of the HC cohort (0.2% when combined) and increased to 0.03–4% of the total abundance of communities in the CF cohort (5% when combined). These OTUs belonged to genera *Escherichia*_*Shigella* (phylum Proteobacteria), *Veilonella*, Megasphaera, *Enterococus, Clostridium XI and Blautia* (phylum Firmicutes).

A number of OTUs, 21 in total, showed relationships with age (F > 5), but could show either 1) no difference or 2) a consistent difference in the abundance between the CF and HC cohorts (Supplementary Figure 4, a subset shown in Fig. 5). This included one of the most abundant OTUs in the dataset associated with the genus *Bacteroides* (phylum Bacteroidetes), which increased in abundance with age, constituting as much as 50% of the community, but showing no difference between the cohorts (Fig. 5a). Another abundant OTU, associated with the genus *Veillonella* (phylum Firmicutes) showed similar decreasing trends with age in both cohorts, but had consistently greater abundance in the CF cohort (Fig. 5b).

### Comparison of OTUs between communities in pancreatic sufficiency cohorts

Only 26 OTUs out of a total of 1721 OTUs passed the abundance filter to justify further analysis. This low pass rate was related to data variability and low sample size. Given the low sample size, we chose only to further investigate differences between cohorts and not trends with age (all ANOVAs: F < 9, P > 0.2) or the interaction between age and cohort (all ANOVAs: F < 5, P > 0.7).

**Figure 6:**
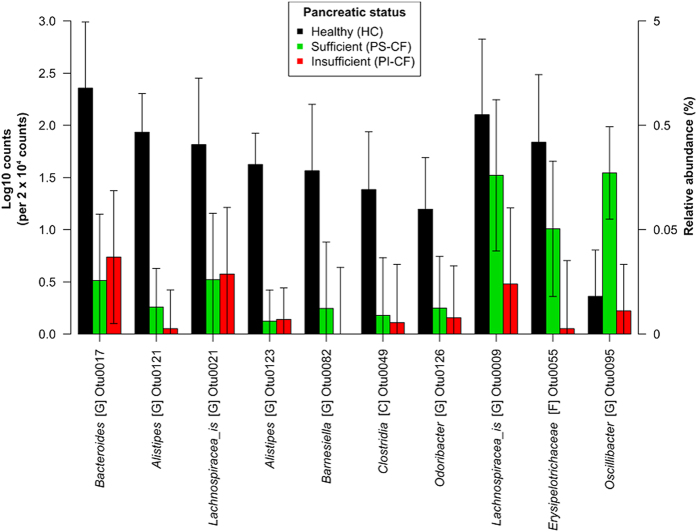
Differentially abundant OTUs in gut microbiomes communities from children with CF and pancreatic sufficiency (PS-CF) or insufficiency (PS-CF), and healthy (HC) children. Mean and 95% confidence intervals are constructed from general linear models (n = 5 for each cohort). Letters in square brackets following taxonomic names denote the lowest level of taxonomic classification: G = genus, F = family, C = class. The suffix “*_is”* = *incertae sedis*.

There were ten OTUs that demonstrated changes associated with the exocrine pancreatic status, each consisting of < 5% of the community (Fig. 6). Seven of the ten OTUs were lower in abundance in both CF cohorts regardless of pancreatic condition. These OTUs were associated with the genera *Bacteroides*, *Alistipes, Lachnospiracea_is* (“*_is”* = *incertae sedis*, uncertain taxonomic placement), *Barnesiella,* as well OTUs associated with the class Clostrida.

Two OTUs were in similar abundance between the HC and CF-PS cohorts, and together in greater abundance compared with the CF-PI cohort. These OTUs belonged to genera *Lachnospiracea_is Erysipelotrichaceae*. Interestingly, there was an OTU associated with the genus *Oscillibacter* that was greater in abundance in the CF-PS cohort compared with the other two cohorts.

## Discussion

Currently there is a lack of detailed information on the changes and progression in diversity and composition of the gut microbiome in children with CF. By comparing the gut microbiota of children with and without CF and across childhood ages, we observed significant effects of both age and cohort (CFTR dysfunction and associated treatments) on the temporal progression of microbial communities. We found that there were specific “imbalances” in the gut microbiota associated with CF, which were already present during the first years of life and could progress farther from the normal path with increasing age. We also identified a number of taxonomic groups that differed in the CF cohort, with contrasting types of abundance changes in comparison to the healthy cohort among childhood. The putative effects of the CF on the gut microbiome i.e. the differences we observed within the CF cohort, could not be disentangled from the effect of antibiotic use within the cohort and the results from our pragmatic approach must be viewed within this context.

Microbial richness and diversity were both significantly reduced in the CF cohort compared with the healthy cohort. The gut microbial richness (total number of bacterial OTUs) increased with age for both healthy and CF cohorts, but was systemically lower in the CF cohort. The microbial richness in the CF cohort within the teenage years did not even reach the same richness found in HC cohort with infancy years. We also observed an increase in microbial diversity (using the Shannon-Weaver index) with age in the gut microbial communities of healthy children, but there was no change in diversity with age in children with CF i.e. the difference in species diversity between the healthy and CF gut microbial communities became larger with age. These observations as well as the gradation in richness and diversity changes from the pancreatic status comparisons (HC > PS-CF > PI-CF), suggest the influence of CFTR dysfunction and associated CF treatments on the gut microbiota affects the natural trajectory of microbial diversity. Previous studies of a murine and ferret gut models of CF reported similar patterns between CF and healthy cohorts^23^,^27^ and linked enrichment of certain bacterial species to loss in CFTR function. Duytschaever, *et al.*^13^ also reported a lower total richness index (TRI, using the number of bands in denaturing gradient gel electrophoresis) for bacterial species in the gut of CF patients compared to controls, but comparisons were made with siblings of CF patients (which we avoided in this study since there is a higher probability of heterozygotes among among affected siblings, who may thus have mild degrees of CFTR dysfunction^28^,^29^,^30^) and no temporal changes in TRI were described. Together, the alpha diversity metrics observed here and in other studies highlight the phenomena of an overall reduced number of bacterial species and an uneven overgrowth by certain bacteria within the gut of children with CF throughout childhood. This lack of diversity is problematic as it has been shown that subjects with increased microbial diversity tended to experience longer time to CF exacerbation and respiratory colonisation by *Pseudomonas aeruginosa*^31^.

Microbial community composition (beta-diversity) showed low similarities within and between cohorts primarily due to changes in composition with age. Importantly, there were strong temporal patterns observed in the healthy cohort, but this pattern was difficult to observe in the CF cohort. The investigation of the change in communities associated with the CF condition has yet to be examined in this context. Other studies have conducted longitudinal studies over 15–20 months of the same subjects^13^, but here we examine a number of children across an age range of 0.87–17 years. In both cases, there were clear reductions in temporal stability of microbial communities associated with CF and thus both short^13^ and long (this study) term instability are now recognised^32^.

In light of the differences found with the alpha- and beta-diversities of the gut microbiota in children with CF, the bacterial species (here, OTUs) that accounted for the differences in abundances with age between CF and HC were investigated (e.g. which bacteria were associated with overgrowth in CF). The most complex changes associated with CF involved deviations from the normal patterns of relative bacterial abundance with age. The relative abundance of a number of OTUs associated with the family *Ruminococcaceae* (phylum Firmicutes) and the genus *Alistipes* (phylum Bacteroidetes) naturally increased with age in healthy children, but not in CF. The patterns in healthy children were generally driven by very low abundances in the early years, which increased with age, while the abundances were consistently low for all ages in CF. This suggests that these microbial groups were unable to establish themselves within the gut microbial communities of children with CF. Members of the *Ruminococcaceae* are well-known butyrate-producers and have been recently described as beneficial for fermentative gut processes in response to prebiotic supplementation^33^. The genus *Alistipes* was also recently proposed to have, in combination with other gut bacteria, a protective role against *Clostridium difficile* infection post antibiotic treatments in a murine gut model^34^. A lack of establishment of such key organisms will thus potentially play a role in long-term gut function and protection against harmful pathogens.

In contrast, we observed *Streptococcus* and *Flavonifractor* OTUs (both phylum Firmicutes) to decrease in abundance with age in the HC cohort, but to remain at a relatively constant abundance across ages in the CF cohort suggesting some early colonisers continued to remain established within the community. In a separate study, *Streptococcus* was reported to be one of the more dominant intestinal bacteria in infants with CF^22^ and this genus was most commonly identified in the lungs of ferrets with CF^27^. *Flavonifractor* is a recently described genus, whose members can convert catechins, a class of bioactive polyphenols abundant in the human diet. Ongoing presence of *Flavonifractor* within the CF gut microbiome might thus considerably affect the proposed health effects of dietary catechins^35^ or be related to maldigestion in CF^36^.

In addition to the differences in abundance trends with age for certain OTUs, there were a number of OTUs that had simple differences in abundances between CF and HC cohorts (i.e. no differential age trends between cohorts). Almost all of these changes occurred within the family *Lachnospiraceae* of the phylum Firmicutes, but the largest decreases were associated with the genera *Alistipes* (see above), *Feacalibacterium* (phylum Firmicutes, family *Ruminococcaceae;* see above) and unclassified genera within family *Erysipelotrichaceae* and order Bacteroidales (Fig. 5). A number of OTUs, associated with genera *Escherichia*_*Shigella* (Proteobacteria), *Enterococcus*, *Veillonella*, *Megasphaera*, *Clostridium* group XI and *Blautia* (all Firmicutes) were also found more abundant in CF compared to HC. The genera *Escherichia*/*Shigella* and *Enterococcus* are well known for their pathogenic and commensal strains, but the phylogenetic resolution of our data (sequence length) did not allow us to define specific strains and traits. *Veillonella* are a group of anaerobic bacteria frequently identified from the airways of children with CF^22^,^37^,^38^ and found to be dominant in the intestine of CF patients^22^. Previous work has suggested a metabolic interaction between the lactic-acid consuming *Veillonella* and lactic-acid producing *Streptococcus*^39^, which correspond to the trends in abundance profile seen here.

Comparison of microbial communities within the CF cohort of children that differed based on CFTR dysfunction severity (pancreatic sufficient or insufficient) showed a slight gradation in similarity from most severe (insufficient) to normal functions (healthy cohort), however, there were greater similarity between the two CF cohorts than when compared with the healthy cohort. This comparison revealed small effects of CFTR dysfunction severity on the gut microbiome but requires further research based on our findings using limited sample size.

This study highlights several important considerations for future clinical translation. In healthy children, the gut microbiota converges toward the characteristic adult profile by the end of the first year of life this study^40^. By 2–3 years old, the microbiota fully resembles that of an adult in terms of composition and diversity this study^41^. Once established, the adult microbiome is stable and relatively difficult to perturb^41^,^42^. We found that gut dysbiosis was not only present in early childhood in CF, but that these changes deviate progressively farther from the path of HC with increasing age. These observations provide a rationale for considering targeted interventions (e.g. probiotics) early rather than later in life in children with CF. Our study also provided insight into possible probiotic strain(s) to use for future therapeutic trial. *Lactobacillus* strains, for instance, demonstrated antagonistic activities against the deleterious effects by potential pathogenic bacteria, such as Escherichia, *Shigella* or *Enterococcus*^43^, which the latter were greatly more abundant in CF subjects compared to HC. Alternatively, members of the *Ruminococcaceae* or genus *Alistipes* could be offered to re-balance metabolic gut process or prevent infection with occasional pathogens, such as *Clostridium difficile.*

Our study however also needs to consider a number of caveats. Firstly, although faecal sampling was performed under strict inclusion and exclusion criteria (see Methods), the majority of CF patients were inevitably exposed to antibiotics (Supplementary table 2), which will affect the microbial composition in the gut. Given our pragmatic approach, it would be difficult to obtain samples free of the influence of antibiotics. What we show here is the condition of the gut microbiota reflecting the life of a child with CF, and indeed, any therapeutic approach would take need to tackle such a scenario. Secondly, a high calorie diet is encouraged for CF patients and it is well established that diet can have significant effects on gut microbiota^44^, but adherence to diet regimes can be highly variable^45^. Thirdly, we could only access and analyse faecal samples, which mainly represent luminal bacteria^46^. Other microbial changes could occur in the epithelial- or mucous-associated communities of gut and this would be important to explore in future studies using intestinal biopsies from CF patients. However, the feasibility of such a study would require the relatively rare clinical indication for gastroduodenoscopy and colonoscopy in children with CF and healthy patients. Lastly, the applicability of our data to other cohorts is difficult given different treatment occur in different countries e.g. the United States do not use prophylactic flucloxacillin as is done in Australia due to problems associated with antibiotic resistance.

In conclusion, it is clear that there are differences in the diversity and composition of gut microbiota between CF and healthy cohorts which manifest themselves with the early years of life. These differences can be observed among the successional changes that occur throughout the childhood years. There was also gradation in change in richness and diversity according to the exocrine pancreatic function. Furthermore, we identified changes in the abundances in gut bacteria in children with CF, with certain OTUs demonstrating either an unchanged, increasing or decreasing pattern with age. Together this work provides further information on the development of the gut microbiome in relation to CF and offers opportunities for the rational design of future treatments to establish a healthy gut microflora.

## Methods

### Sampling of subjects

Children aged from 0–18 years old from the CF clinic at the Sydney Children’s Hospital Randwick were enrolled prospectively. Children were diagnosed with CF according to the United States Cystic Fibrosis Foundation consensus criteria^47^. Patients with a pulmonary exacerbation (in the preceding four weeks before sampling) requiring intravenous antibiotics were excluded. Patients on oral antibiotics in the preceding four weeks, other than oral prophylaxis against *Staphylococcus aureus* (flucloxacillin), *Haemophilus influenzae* (amoxicillin or amoxicillin-clavulanate), and *Pseudomonas aeruginosa* (azithromycin) were also excluded. The exocrine pancreatic function status, defined based on the 72-hour faecal fat and/or faecal elastase-1^48^,^49^, was also recorded and further grouped the CF cohort into pancreatic insufficient (PI-CF) or pancreatic sufficient (PS-CF).

Children without CF, inflammatory bowel disease (IBD) or gastrointestinal complaints were prospectively recruited as healthy controls (HC). Any subject (from either CF or HC cohort) with gastroenteritis, on oral corticosteroids, probiotics and/or non-steroidal anti-inflammatory drugs in the preceding two weeks were excluded. One faecal sample was collected from each subject. Samples were stored −80 °C if the stool was collected on the same day as submission to the laboratory, or stored at −20 °C (home freezer) until transport to the laboratory, where they were then stored at −80 °C. Thawing of sample did not occur during transport and all samples were processed together for microbial analysis.

Written informed consent was obtained from each subject or caregiver(s). The study was approved by the South Eastern Sydney Area Health Service, Human Research Ethics Committee, Sydney, Australia (HREC ref no: 10/240) and carried out in accordance with the approved guidelines.

### DNA extraction and sequencing of the gut microbial community 16S rRNA genes

Genomic DNA was extracted from homogenised stool samples using QIAamp DNA Mini Kit (Qiagen) following manufacturer’s instructions. The DNA was checked for quality and quantity using gel electrophoresis. Community 16S rRNA genes were amplified with the primers 27F (AGAGTTTGATCMTGGCTCAG) and 519R (GWATTACCGCGGCKGCTG) spanning the V1–V3 gene regions and sequenced using the Illumina MiSeq platform (v3, 2 × 300bp). The forward reads (300 bp) were used to examine the V1–V2 regions.

Sequences were quality filtered using Mothur^50^ following the MiSeq SOP (without contig formation and instead using trim.seqs with qwindowsize = 5, qwindowaverage = 30, minlength = 100, maxambig = 0, maxhomop = 8)^51^ and clustered into operational taxonomic units (OTUs) using 97% sequence similarity. OTUs were classified using the Ribosomal Database Project (RDP) taxonomic outline version 9^52^, with a 60% confidence cut off. An uneven sequencing depth was observed among samples as a result of sample pooling and sequencing in one run. Samples with <20 008 sequences per sample were removed, with this threshold based on the distribution of the number of sequences obtained per sample and the trade-off between sequencing depth and number of samples for the comparing of between HC and CF groups. The study started with 49 CF (43 PI-CF and 6 PS-CF) and 40 HC samples, and was left with 23 CF (20 PI-CF, 3 PS-CF) and 35 HC samples after removal of ‘defective’ samples. The spread of childhood ages between the HC and CF cohorts of the remaining samples were similar and resulted in age continuing to be a satisfactory covariate in our analysis (Supplementary Figure 1). The number of sequences per sample was equalised between samples (n = 20 008) by random subsampling to remove the effect of differential sequencing depth among samples.

This sequence filter above, however, resulted in a low number of PS-CF samples precluding statistically meaningful comparison of exocrine pancreatic status. Therefore, a second filtered dataset was generated with a threshold of 9751 sequence per sample. This resulted in n = 5 for PS-CF cohort, which were compared to five closest age-matched samples each from the HC and PI-CF cohorts. Thus two separate datasets were used in the study: (1) comparisons between CF and HC (sequencing depth per sample = 20 008), and (2) comparisons based on exocrine pancreatic status (HC vs. PS-CF vs. PI-CF, sequencing depth per sample = 9751, see below).

### Data analysis

Comparisons of microbial alpha- and beta-diversities were conducted to determine differences associated with the CF disease (i.e. HC vs. CF) and exocrine pancreatic function (i.e. HC vs. PS-CF vs. PI-CF) as well as trends associated with age. The microbial species diversity (alpha-diversity) was examined using two metrics including the number of OTUs (a measure of richness) and the Shannon-Weaver index (a measure of diversity). The Shannon-Weaver index takes into account the different types of species (here, OTUs) as well as how evenly distributed the species are within a sample. The index increases as the number of species and the evenness between them increases. General linear models were constructed to examine the effects of the various predictors including age (continuous covariate), CF disease (categorical covariate) and exocrine pancreatic status (categorical covariate) on the alpha diversity metrics using the R package CAR^53^. Models were checked via visualisation of residual plots and transformations were conducted, if they resulted in better model fits. Significance of model terms was examined using ANOVA with type II SS and an alpha level of 5% (Supplementary table 1). Non-significant model terms were removed before extraction of model estimates and confidence intervals.

Microbial communities (beta-diversity) were compared using two approaches: 1) distance-based comparisons (considered ‘community level comparisons’) and 2) comparison of each OTU separately. For the distance-based approach, OTU relative abundances were square-root transformed and the Bray-Curtis similarity coefficient calculated between each and every sample pair. The similarity matrix was visualised using non-metric multidimensional scaling (nMDS). PERMANOVA was used to test for significance of model terms using type II SS and an alpha level of 5% (Supplementary table 1). Distance-based analysis was conducted using the software package PRIMER version 6^54^. For the comparison of each OTU, general linear models were constructed to examine the effects of the various predictors (as above) on the abundance of each OTU using the R package MVAbund^55^. An abundance filter was used to remove OTUs that were likely of little interest due to low counts across the data set and overcome the high variability in OTUs counts. We chose to remove OTUs, where abundance counts of >20 (0.1% of the total, below which is considered rare^56^) were not observed in at least 75% of the samples before comparison. Sequence counts were Log10 + 1 transformed, which improved the distribution of the data towards normality. P-values were obtained using 999 bootstraps of residuals and adjusted for the number of comparisons made using methods within MVAbund^55^.

## Additional Information

**How to cite this article**: Nielsen, S. *et al.* Disrupted progression of the intestinal microbiota with age in children with cystic fibrosis. *Sci. Rep.* **6**, 24857; doi: 10.1038/srep24857 (2016).

## Supplementary Material

Supplementary Information

## References

[b1] Riordan J. R. *et al.* Identification of the cystic fibrosis gene: cloning and characterization of complementary DNA. Science 245, 1066–1073, doi: 10.1126/science.2475911 (1989). 2475911

[b2] Riordan J. R. CFTR function and prospects for therapy. Annu. Rev. Biochem. 77, 701–726, doi: 10.1146/annurev.biochem.75.103004 (2008). 18304008

[b3] Ahmed N. *et al.* Molecular consequences of cystic fibrosis transmembrane regulator (CFTR) gene mutations in the exocrine pancreas. Gut 52, 1159–1164, doi: 10.1136/gut.52.8.1159 (2003). 12865275 PMC1773762

[b4] Ooi C. Y. & Durie P. R. Cystic fibrosis transmembrane conductance regulator (CFTR) gene mutations in pancreatitis. J. Cyst. Fibr 11, 355–362, doi: 10.1016/j.jcf.2012.05.001 (2012). 22658665

[b5] Ooi C. Y. *et al.* Type of CFTR mutation determines risk of pancreatitis in patients with cystic fibrosis. Gastroenterology 140, 153–161, doi: 10.1053/j.gastro.2010.09.046 (2011). 20923678

[b6] Garcia M. A. S., Yang N. & Quinton P. M. Normal mouse intestinal mucus release requires cystic fibrosis transmembrane regulator–dependent bicarbonate secretion. J. Clin. Invest. 119, 2613, doi: 10.1172/JCI38662 (2009). 19726884 PMC2735925

[b7] Gelfond D., Ma C., Semler J. & Borowitz D. Intestinal pH and gastrointestinal transit profiles in cystic fibrosis patients measured by wireless motility capsule. Dig. Dis. Sci. 58, 2275–2281, doi: 10.1007/s10620-012-2209-1 (2013). 22592630

[b8] Stoltz D. A. *et al.* Cystic fibrosis pigs develop lung disease and exhibit defective bacterial eradication at birth. Sci. Transl. Med. 2, 29ra31, doi: 10.1126/scitranslmed.3000928 (2010). PMC2889616 20427821

[b9] Ooi C. Y. *et al.* Fecal Human β-Defensin 2 in Children with Cystic Fibrosis: Is There a Diminished Intestinal Innate Immune Response? Dig. Dis. Sci. 60, 1–7, doi: 10.1007/s10620-015-3842-2 (2015). 26271615

[b10] O’Brien S. *et al.* Intestinal bile acid malabsorption in cystic fibrosis. Gut 34, 1137–1141, doi: 10.1136/gut.34.8.1137 (1993). 8174969 PMC1374370

[b11] Bruzzese E. *et al.* Disrupted intestinal microbiota and intestinal inflammation in children with cystic fibrosis and its restoration with Lactobacillus GG: a randomised clinical trial. PLoS One 9, e87796, doi: 10.1371/journal.pone.0087796 (2014). 24586292 PMC3929570

[b12] Duytschaever G. *et al.* Dysbiosis of bifidobacteria and Clostridium cluster XIVa in the cystic fibrosis fecal microbiota. J. Cyst. Fibr 12, 206–215, doi: 10.1016/j.jcf.2012.10.003 (2013). 23151540

[b13] Duytschaever G. *et al.* Cross-sectional and longitudinal comparisons of the predominant fecal microbiota compositions of a group of pediatric patients with cystic fibrosis and their healthy siblings. Appl. Environ. Microbiol. 77, 8015–8024, doi: 10.1128/AEM.05933-11 (2011). 21926193 PMC3208981

[b14] Scanlan P. D. *et al.* Gut dysbiosis in cystic fibrosis. J. Cyst. Fibr 11, 454–455, doi: 10.1016/j.jcf.2012.03.007 (2012). 22538067

[b15] Schippa S. *et al.* Cystic fibrosis transmembrane conductance regulator (CFTR) allelic variants relate to shifts in faecal microbiota of cystic fibrosis patients. PLoS One 8, 1176, doi: 10.1371/journal.pone.0061176 (2013). PMC3629184 23613805

[b16] Gill S. R. *et al.* Metagenomic analysis of the human distal gut microbiome. Science 312, 1355–1359, doi: 10.1126/science.1124234 (2006). 16741115 PMC3027896

[b17] Turnbaugh P. J. *et al.* An obesity-associated gut microbiome with increased capacity for energy harvest. Nature 444, 1027–1131, doi: 10.1038/nature05414 (2006). 17183312

[b18] Lee J. M. *et al.* Update of faecal markers of inflammation in children with cystic fibrosis. Mediators Inflamm. 2012, 948367, doi: 10.1155/2012/948367 (2012). 22988347 PMC3439990

[b19] Dhaliwal J. *et al.* Intestinal Inflammation and Impact on Growth in Children With Cystic Fibrosis. J. Pediatr. Gastroenterol. Nutr. 60, 521–526, doi: 10.1097/MPG.0000000000000683. (2015). 25539196

[b20] Flass T. *et al.* Intestinal Lesions Are Associated with Altered Intestinal Microbiome and Are More Frequent in Children and Young Adults with Cystic Fibrosis and Cirrhosis. PLoS One 10, e0116967, doi: 10.1371/journal.pone.0116967 (2015). 25658710 PMC4319904

[b21] Norkina O., Burnett T. G. & De Lisle R. C. Bacterial overgrowth in the cystic fibrosis transmembrane conductance regulator null mouse small intestine. Infect. Immun. 72, 6040–6049, doi: 10.1128/IAI.72.10.6040-6049.2004 (2004). 15385508 PMC517588

[b22] Madan J. *et al.* Serial analysis of the gut and respiratory microbiome in cystic fibrosis in infancy: interaction between intestinal and respiratory tracts and impact of nutritional exposures. mBio 3, e00251–00212, doi: 10.1128/mBio.00251-12 (2012). 22911969 PMC3428694

[b23] Lynch S. V. *et al.* Cystic fibrosis transmembrane conductance regulator knockout mice exhibit aberrant gastrointestinal microbiota. Gut Micro. 4, 41–47, doi: 10.4161/gmic.22430 (2013). PMC3555885 23060053

[b24] Schippa S. *et al.* Cystic fibrosis transmembrane conductance regulator (CFTR) allelic variants relate to shifts in faecal microbiota of cystic fibrosis patients. PloS one 8, e61176 (2013). 23613805 10.1371/journal.pone.0061176 PMC3629184

[b25] Koenig J. E. *et al.* Succession of microbial consortia in the developing infant gut microbiome. Proc. Natl. Acad. Sci. USA 108, 4578–4585, doi: 10.1073/pnas.1000081107 (2011). 20668239 PMC3063592

[b26] Thompson-Chagoyán O. C., Maldonado J. & Gil A. Colonization and impact of disease and other factors on intestinal microbiota. Dig. Dis. Sci. 52, 2069–2077, doi: 10.1007/s10620-006-9285-z (2007). 17420934

[b27] Sun X. *et al.* Gastrointestinal pathology in juvenile and adult CFTR-knockout ferrets. Am. J. Pathol. 184, 1309–1322, doi: 10.1016/j.ajpath.2014.01.035 (2014). 24637292 PMC4005986

[b28] Ooi C. Y. *et al.* Does integration of various ion channel measurements improve diagnostic performance in cystic fibrosis? Ann. American. Thorac. Soc. 11, 562–570, doi: 10.1513/AnnalsATS.201311-412OC (2014). 24697796

[b29] Ooi C. Y. *et al.* Does extensive genotyping and nasal potential difference testing clarify the diagnosis of cystic fibrosis among patients with single-organ manifestations of cystic fibrosis? Thorax 69, 254–260, doi: 10.1136/thoraxjnl-2013-203832 (2014). 24149827

[b30] Wilschanski M. *et al.* Mutations in the cystic fibrosis transmembrane regulator gene and *in vivo* transepithelial potentials. Am. J. Respir. Crit. Care Med. 174, 787–794, doi: 10.1164/rccm.200509-1377OC (2006). 16840743 PMC2648063

[b31] Hoen A. G. *et al.* Associations between gut microbial colonization in early life and respiratory outcomes in cystic fibrosis. J. Pedia. 167, 138–147. e133, doi: 10.1016/j.jpeds.2015.02.049 (2015). PMC4674690 25818499

[b32] Huang Y. J. & Lynch S. V. The emerging relationship between the airway microbiota and chronic respiratory disease: clinical implications. Expert Rev. Respir. Med. 5, 809–821, doi: 10.1586/ers.11.76 (2011). 22082166 PMC3359942

[b33] Scott K. P., Martin J. C., Duncan S. H. & Flint H. J. Prebiotic stimulation of human colonic butyrate-producing bacteria and bifidobacteria, *in vitro*. FEMS Microbiol. Ecol. 87, 30–40, doi: 10.1111/1574-6941.12186 (2014). 23909466

[b34] Schubert A. M., Sinani H. & Schloss P. D. Antibiotic-induced alterations of the murine gut microbiota and subsequent effects on colonization resistance against Clostridium difficile. mBio 6, e00974–00915, doi: 10.1128/mBio.00974-15 (2015). 26173701 PMC4502226

[b35] Kutschera M., Engst W., Blaut M. & Braune A. Isolation of catechin‐converting human intestinal bacteria. J. Appl. Microbiol. 111, 165–175, doi: 10.1111/j.1365-2672.2011.05025 (2011). 21457417

[b36] Vaisman N., Tabachnik E. & Sklan D. Short-chain fatty acid absorption in patients with cystic fibrosis. J. Pediatr. Gastroenterol. Nutr. 15, 146–149 (1992). 1403462 10.1097/00005176-199208000-00008

[b37] Brook I. & Fink R. Transtracheal aspiration in pulmonary infection in children with cystic fibrosis. Eur. J. Respir. Dis. 64, 51–57, doi: 6825749 (1983). 6825749

[b38] Tunney M. M. *et al.* Detection of anaerobic bacteria in high numbers in sputum from patients with cystic fibrosis. Am. J. Respir. Crit. Care Med. 177, 995–1001, doi: 10.1164/rccm.200708-1151OC (2008). 18263800

[b39] van den Bogert B. *et al.* Diversity of human small intestinal Streptococcus and Veillonella populations. FEMS Microbiol. Ecol. 85, 376–388, doi: 10.1111/1574-6941.12127 (2013). 23614882

[b40] Palmer C., Bik E. M., DiGiulio D. B., Relman D. A. & Brown P. O. Development of the human infant intestinal microbiota. PLoS Biol. 5, e177, doi: 10.1371/journal.pbio.0050177 (2007). 17594176 PMC1896187

[b41] Yatsunenko T. *et al.* Human gut microbiome viewed across age and geography. Nature 486, 222–227, doi: 10.1038/nature11053 (2012). 22699611 PMC3376388

[b42] Faith J. J. *et al.* The long-term stability of the human gut microbiota. Science 341, 1237439, doi: 10.1126/science.1237439 (2013). 23828941 PMC3791589

[b43] Reid G. The scientific basis for probiotic strains of Lactobacillus. Appl. Environ. Microbiol. 65, 3763–3766 (1999). 10473372 10.1128/aem.65.9.3763-3766.1999 PMC99697

[b44] David L. A. *et al.* Diet rapidly and reproducibly alters the human gut microbiome. Nature 505, 559–563, doi: 10.1038/nature12820 (2014). 24336217 PMC3957428

[b45] Kawchak D. A. *et al.* Longitudinal, prospective analysis of dietary intake in children with cystic fibrosis. J. Pedia. 129, 119–129, doi: 10.1016/S0022-3476(96)70198-1 (1996). 8757571

[b46] Zoetendal E. G. *et al.* Mucosa-Associated Bacteria in the Human Gastrointestinal Tract Are Uniformly Distributed along the Colon and Differ from the Community Recovered from Feces. Appl. Environ. Microbiol. 68, 3401–3407, doi: 10.1128/aem.68.7.3401-3407.2002 (2002). 12089021 PMC126800

[b47] Farrell P. M. *et al.* Guidelines for diagnosis of cystic fibrosis in newborns through older adults: Cystic Fibrosis Foundation consensus report. J. Pedia. 153, S4–S14, doi: 10.1016/j.jpeds.2008.05.005 (2008). PMC2810958 18639722

[b48] Jeejeebhoy K., Ahmad S. & Kozak G. Determination of fecal fats containing both medium and long chain triglycerides and fatty acids. Clin. Biochem. 3, 157–163, doi: 10.1016/S0009-9120(70)80021-2 (1970). 5527090

[b49] Löser C., Möllgaard A. & Fölsch U. Faecal elastase 1: a novel, highly sensitive, and specific tubeless pancreatic function test. Gut 39, 580–586, doi: 10.1136/gut.39.4.580 (1996). 8944569 PMC1383273

[b50] Schloss P. D. *et al.* Introducing mothur: open-source, platform-independent, community-supported software for describing and comparing microbial communities. Appl. Environ. Microbiol. 75, 7537–7541, doi: 10.1128/AEM.01541-09 (2009). 19801464 PMC2786419

[b51] Kozich J. J., Westcott S. L., Baxter N. T., Highlander S. K. & Schloss P. D. Development of a dual-index sequencing strategy and curation pipeline for analyzing amplicon sequence data on the MiSeq Illumina sequencing platform. Appl. Environ. Microbiol. 79, 5112–5120, doi: 10.1128/AEM.01043-13 (2013). 23793624 PMC3753973

[b52] Cole J. R. *et al.* The Ribosomal Database Project: improved alignments and new tools for rRNA analysis. Nucleic Acids Res. 37, D141–D145, doi: 10.1093/nar/gkn879 (2009). 19004872 PMC2686447

[b53] Fox J. & Weisberg S. An R Companion to Applied Regression. 2 edn, (Sage, 2011).

[b54] Clarke K. & Gorley R. PRIMER v6 User manual/tutorial. Plymouth routine in mulitvariate ecological research. Plymouth Marine Laboratory. (PRIMER-E Ltd, 2006).

[b55] Wang Y., Naumann U., Wright S. T. & Warton D. I. mvabund–an R package for model‐based analysis of multivariate abundance data. Methods Ecol. Evol. 3, 471–474, doi: 10.1111/j.2041-210X.2012.00190.x (2012).

[b56] Lynch M. D. J. & Neufeld J. D. Ecology and exploration of the rare biosphere. Nat. Rev. Microbiol. 13, 217–229, doi: 10.1038/nrmicro3400 (2015). 25730701

