# Supplementary material for: Disrupted progression of the intestinal microbiota with age in children with cystic fibrosis
Source: Sci Rep. 2016 May 4;6:24857. doi: 10.1038/srep24857 (PMC4855157; doi:10.1038/srep24857)
Supplement: Supplementary Information [file srep24857-s1.pdf]

Supplementary material for:

Disrupted progression of the intestinal microbiota with age in children with cystic fibrosis

Shaun Nielsen*[1,2], Bronwen Needham[2], Steven T. Leach[3], Andrew S. Day[4], Adam Jaffe[3,5], Torsten Thomas[1,2], Chee Y. Ooi*[3,6].

[1]Centre for Marine Bio-innovation, [2]School of Biotechnology and Biomolecular Sciences, and [3]Discipline of Paediatrics, School of Women's and Children's Health, Medicine, University of New South Wales, Sydney, 2052, Australia

[4]Department of Paediatrics, University of Otago, Christchurch, 8011, New Zealand

[5]Department of Paediatric Respiratory and [6]Department of Paediatric Gastroenterology, Sydney Children's Hospital, Randwick, 2031, Australia

* Corresponding authors

E-mail: s.nielsen@unsw.edu.au, keith.ooi@hongyee.org

Tel: +61 2 9385 2741

Supplementary figures.

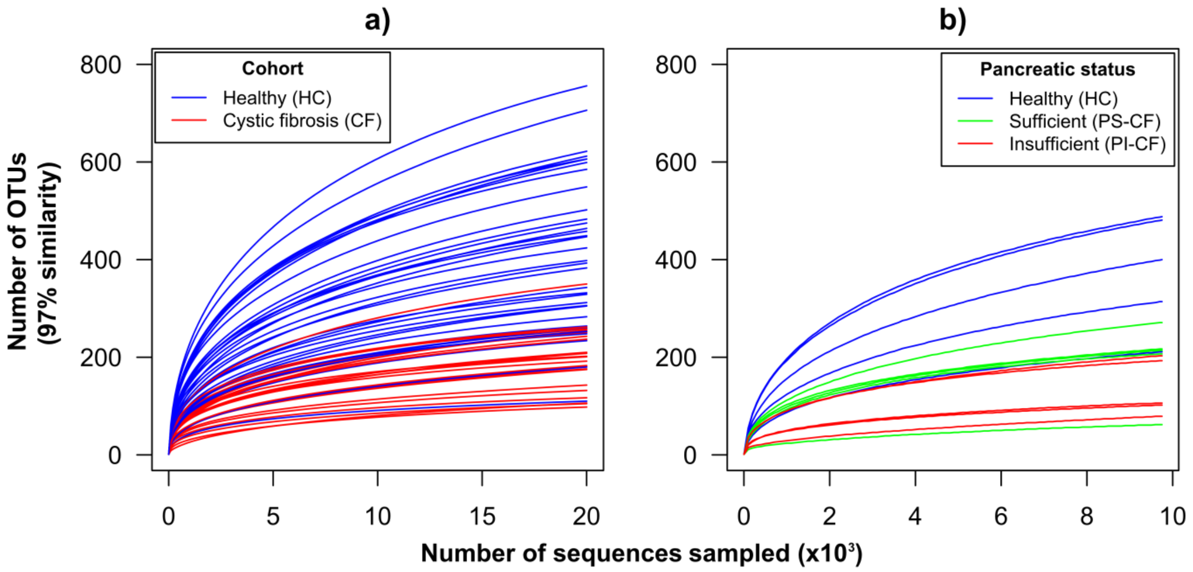

Supplementary Figure 1. The number of OTUs (97 % similarity) given the number of 16S rRNA gene sequences sampled per stool sample. a) samples from healthy vs CF comparisons with 20 008 total sequences per stool, b) samples from the pancreatic status comparison with 9751 total sequences per stool sample.

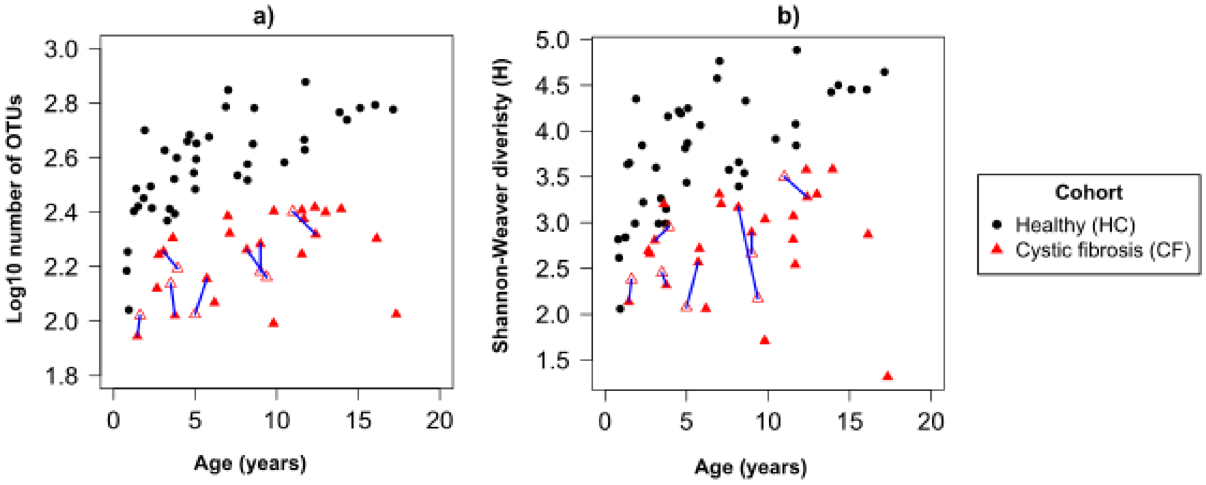

Supplementary Figure 2. Within-subject variability of a) the Log10 number of OTUs and b) the Shannon-Weaver index of diversity with the CF cohort. Subjects are connected by blue lines with the second sample symbol unfilled, and show within-variability is within the normal variability observed within the cohort for both alpha-diversity measures.

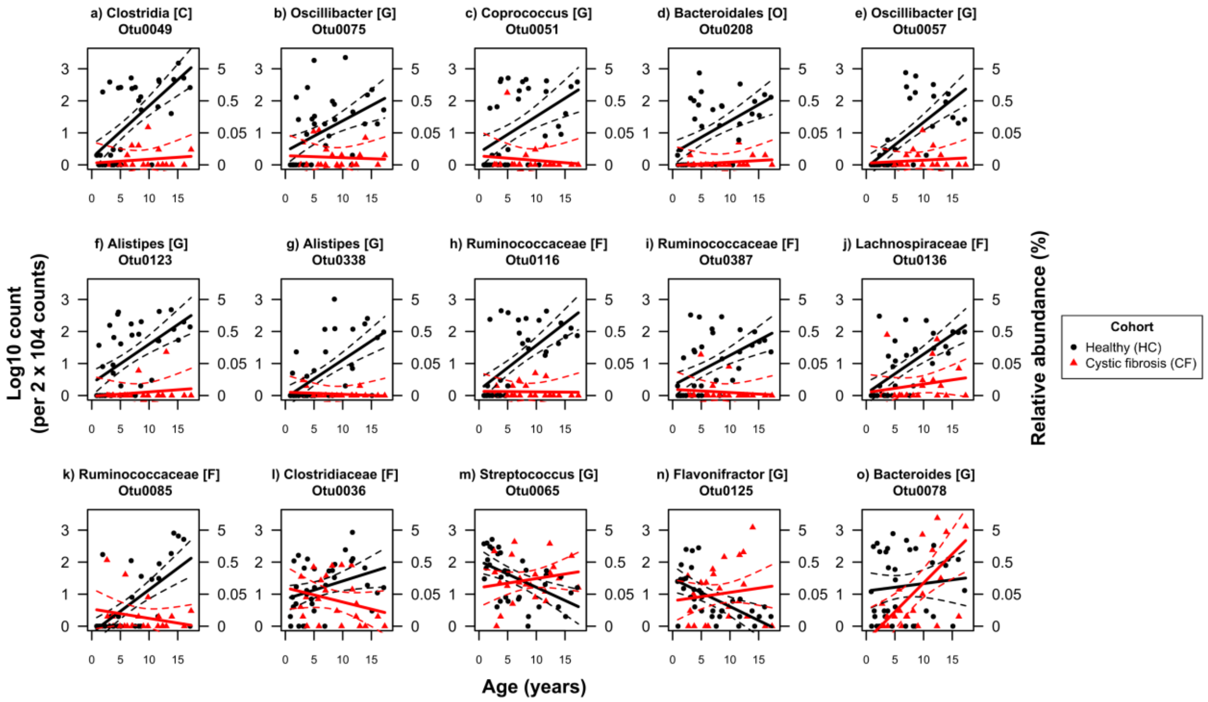

Supplementary Figure 3. Patterns of abundances of OTUs showing interactions between the covariates age and CF condition (based on F values > 5). The figure shows the different relationship types between age and the abundance of OTUs in gut microbiomes communities from CF and healthy (HC) children. Fitted lines are constructed from general linear models. Letters in square brackets following taxonomic names denote the lowest level of taxonomic classification, G = genus, F = family, O = order, C = class.

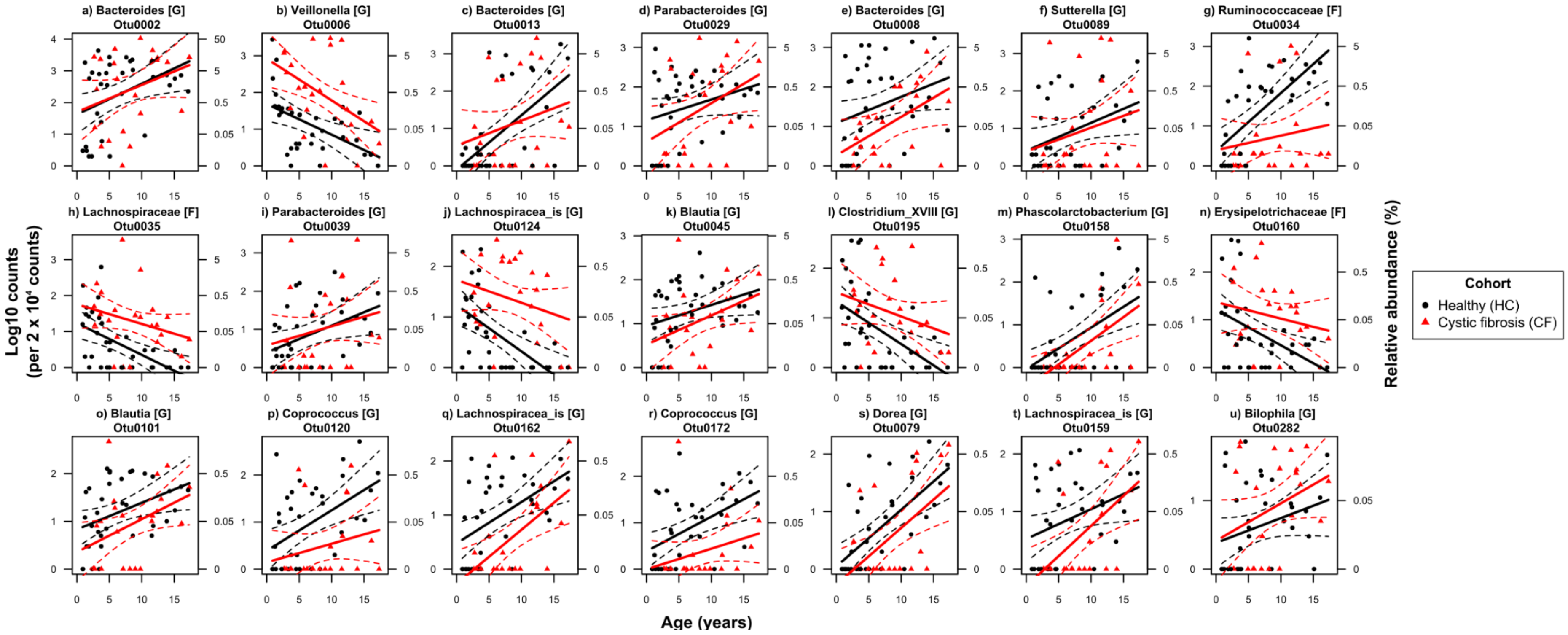

Supplementary Figure 3. Patterns of abundances of OTUs showing relationships with the covariates age and CF condition (based on F values > 5). The figure shows similar relationships between age and the abundance of OTUs in gut microbiomes communities from CF and healthy (HC) children. Fitted lines are constructed from general linear models. Letters in square brackets following taxonomic names denote the lowest level of taxonomic classification, G = genus, F = family, O = order, C = class.

Supplementary tables.

Sample	Included_in_analysis	Treatment	SampleTime	Age	ID	FEV1%predicted	**Tobramycin (nebulised)**	**Piperacillin**	**colomycin/ Colistin**	**Itraconazole**	**Fluconazol**	**prednisolone**	**Azithromycin**	**Flucloxacillin/ Flopen**	**Augmentin Duo**	**amoxycillin**	1. Antibiotics	2a. S. aureus prophylaxs	2b. S. aureus prophylaxis (0-3/5 year vs Long-term)	3. H. influenzae	4. Pseudomonas aeruginosa	5a. Other microorganisms	5b. Specify 5a
14	Y	CF	1	2.66	50a		.	.	.	.	.	.	.	o	.	.	o	o	o	.	.	o	Commensals
26	Y	CF	1	2.76	41a		o	.	.	.	.	.	.	o	.	.	o						
35	Y	CF	1	3.06	48a		.	.	.	.	.	.	.	o	.	.	o	o		o	o	o	Commensals
21	Y	CF	1	3.63	52b		.	.	.	.	.	.	.	.	.	.	o	o	o	o	.	o	Mycobacteriun avium intercellulare
50	Y	CF	1	3.76	47b		.	.	.	.	.	.	.	o	.	.	o	o	o	o	o	o	Commensals
76	Y	CF	1	4.93	71a	96	.	.	.	.	.	.	o	.	.	.	o						
24	Y	CF	1	5.72	43a	97	.	.	.	.	.	.	o	.	.	.	o	o	o	o	o	o	Commensals
2	Y	CF	1	5.86	3b	93	.	.	.	.	.	.	.	.	.	.	o	o	o	o	.	o	Branhamella catarrhalis; aspergillus fumigatus
19	Y	CF	1	6.19	44a	93	.	.	.	.	.	.	.	o	.	.	o	o	.	o	o	o	S. pneumoniae
56	Y	CF	1	7	12a	97	.	.	.	.	.	.	.	o	.	.	o	o	.	o	.	o	Commensals
23	Y	CF	1	7.12	35b	11	.	.	.	.	.	.	.	o	.	.	o	o	o	o	o	o	Commensals; aspergillus fumigatus; klebsiella species
84	Y	CF	1	8.17	32a	86	.	.	.	.	.	.	.	o	.	.	o	o	.	.	o	o	Commensals; coliform
52	Y	CF	1	9	15a	64	.	.	.	.	.	.	.	o	.	.	o	o	o	o	o	o	Enterobacter sp.; commensals; Streptococcus; E. coli;
78	Y	CF	1	9.8	27b	86	o	.	.	.	.	.	o	o	.	.	o						
41	Y	CF	1	9.82	34b	10	.	.	.	.	.	.	.	o	.	.	o	o	.	o	.	o	E. coli; aspergillus fumigatus
20	Y	CF	1	11.5	8b	11	.	.	.	.	.	.	.	o	.	.	o	o	.	.	o	o	E. coli; Rhizopus sp.; MRSA; aspergillus fumigatus
74	Y	CF	1	11.6	16b	10	.	.	.	.	.	.	.	o	.	.	o	o	.	o	o	o	Enterobacter sp.; commensals; branhamella catarrhalis
70	Y	CF	1	12.3	28a	84	.	.	.	.	.	.	.	.	.	.	o	o	.	o	.	o	S. pneumoniae; branhamella catarrhalis
43	Y	CF	1	12.4	30b	99	.	.	.	.	.	.	.	o	.	.	o	o	o	.	o	o	Candida albicans; RSV
47	Y	CF	1	13	14a	88	o	.	.	.	.	.	o	.	.	.	o	.	.	o	o	o	Aspergillus fumigatus
34	Y	CF	1	13.9	7b												o						
66	Y	CF	1	16.1	26a	72	.	.	.	.	.	.	.	o	.	.	o	o	.	.	o	.	
27	Y	CF	1	17.3	10b	12	o	.	.	.	.	.	o	o	.	.	o						
72	Y	CF	3	1.46	51a		.	.	.	.	.	.	.	o	.	.	o	o		o	.	o	Aspergillus fumigatus
71	N	CF	2	9	15b												o	o	o	o	o	o	Enterobacter sp.; commensals; Streptococcus; E. coli;
86	N	CF	2	11	30a	10	.	.	.	.	.	.	.	o	.	.	o	o	o	.	o	o	Candida albicans; RSV
87	N	CF	2	9.38	32b	95	.	.	.	.	.	.	o	.	.	.	o	o	.	.	o	o	Commensals; coliform
51	N	CF	2	5	35a	10	.	.	.	.	.	.	.	o	.	.	o	o	o	o	o	o	Commensals; aspergillus fumigatus; klebsiella species
90	N	CF	2	3.5	47a		.	.	.	.	.	.	.	o	.	.	o	o	o	o	o	o	Commensals
45	N	CF	2	3.92	48b		o	.	.	.	.	.	.	o	.	.	o	o		o	o	o	Commensals
89	N	CF	2	1.63	51b		.	.	.	.	.	.	.	o	.	.	o	o		o	.	o	Aspergillus fumigatus

Supplementary Table 1. Antibiotic use within the CF cohort (o = Yes and . = No, empty cells = No data)
